# Metachronous Gastrointestinal Stromal Tumors of Different Histologies: An Unusual Case

**DOI:** 10.7759/cureus.59159

**Published:** 2024-04-27

**Authors:** Richard Nudotor, Abdel-Moneim M Ali, Adam Weltz, Adrian Park, Glen Gibson

**Affiliations:** 1 Surgery, Anne Arundel Medical Center, Annapolis, USA

**Keywords:** gastrointestinal stromal tumor, jejunum, gastric, epithelial, spindle, gist

## Abstract

Gastrointestinal stromal tumors (GISTs) are mesenchymal tumors accounting for only a small fraction of all primary malignant tumors of the gastrointestinal tract. Histologically, GISTs are classified as epithelioid, spindle type, or mixed. We present a case of a 66-year-old male incidentally noted to have a pedunculated gastric mass along the lesser curvature of the stomach during a laparoscopic Nissen fundoplication and hiatal hernia repair. A wedge resection was performed and the pathology demonstrated a 3.7 cm GIST of epithelioid type. Four years after the initial surgery, a jejunal mass was identified via CT enterography as part of a workup for ongoing iron deficiency anemia. A laparoscopic small bowel resection was performed, and the pathology revealed a new primary 3.2 cm GIST of the spindle cell subtype. Three years after surgery, surveillance imaging is negative for any recurrence. This appears to be the first report of the occurrence of metachronous primary GISTs of different histologic subtypes, separated by location.

## Introduction

Gastrointestinal stromal tumors (GISTs) are mesenchymal tumors accounting for only about 1% of all primary malignant tumors of the gastrointestinal tract [1]. GISTs have an incidence of ~1.2 per 105 individuals per year [2]. Histologically, GISTs are classified as spindle cell type (70%), epithelioid cell type (20%), or mixed (10%) [3]. Of GISTs located in the stomach, there is a nearly equal incidence of epithelioid vs. spindle cell subtypes, while the vast majority of extragastric GISTs are of the spindle cell subtype (90%). Reports have suggested that the epithelioid subtype GISTs have a greater propensity for aggressive behavior (larger size, elevated mitotic index, and higher rates of metastasis) [4].

Treatment of GISTs entails surgical resection when possible, as well as treatment with targeted systemic agents such as imatinib [5]. Prognosis correlates with tumor size, mitotic rate, and the presence of metastatic disease, with five-year survival greater than 90% with small, localized tumors and dropping to below 50% with the presence of metastatic disease [6]. While local recurrence and metastatic disease development in GISTs are well-documented in the medical literature, our case represents the first documented instance, to our knowledge, of two metachronous primary GISTs presenting with different histologic subtypes in the same patient over a period of four years. Remarkably, both cases have been successfully managed, with no evidence of recurrence observed three years following the treatment of the second tumor. This unique case underscores the variability in the clinical course of GISTs and highlights the efficacy of vigilant long-term surveillance and individualized management strategies in achieving favorable outcomes.

## Case presentation

**Figure 1:**
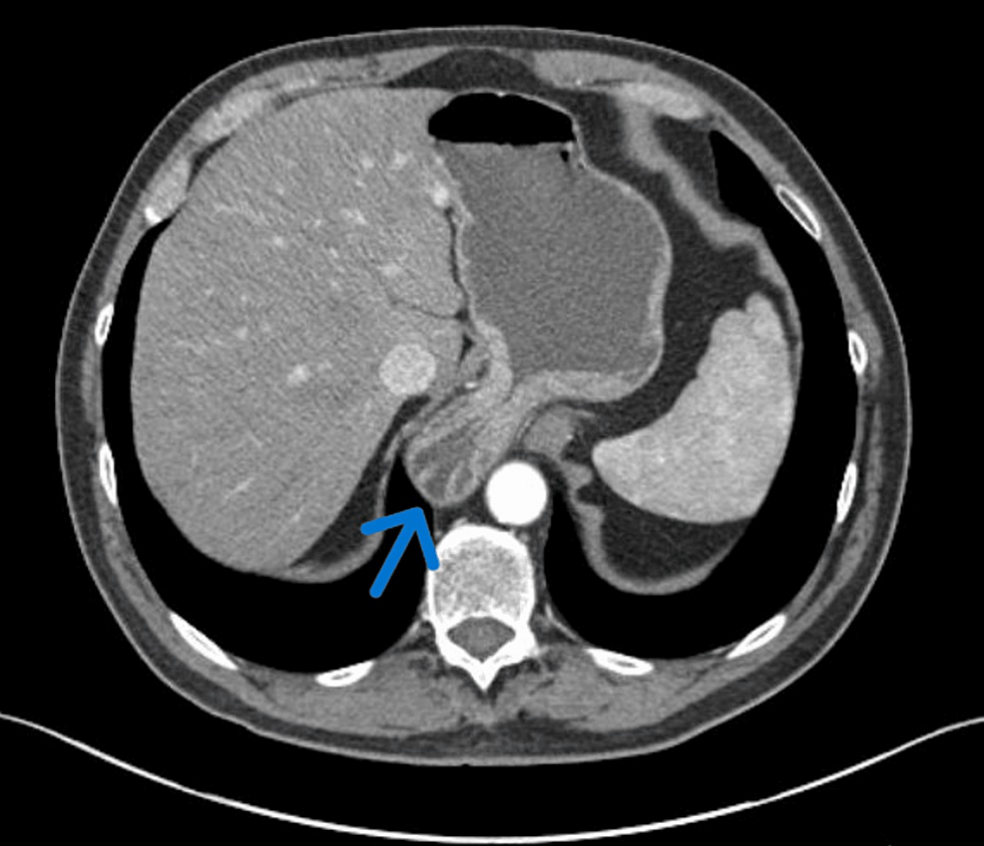
CT enterography showing a hiatal hernia without any evidence of a gastric mass.

A 66-year-old male with a history of gastroesophageal reflux disease and Barrett’s esophagus was referred to the minimally invasive surgery service of Anne Arundel Medical Center for surgical intervention for a large type III paraesophageal hernia. Preoperative workups, including upper endoscopy and CT enterography, were unremarkable except for a large hiatal hernia (Figure 1).

Intraoperatively, a small pedunculated mass was noted along the lesser curvature of the stomach and a wedge resection was performed. The pathology demonstrated epithelioid GIST, 3.7 cm in greatest dimension and with less than five mitosis per 50 high power field. No areas of necrosis were identified. Immunostaining revealed that C-KIT and DOG1 were immunoreactive, and the tumor was considered a low-risk lesion. Imaging studies, including CT scans of the abdomen and pelvis, showed no evidence of lymph node involvement or distant metastasis (the American Joint Committee on Cancer (AJCC) 8th edition, pT2N0M0, Stage I) [7].

**Figure 2:**
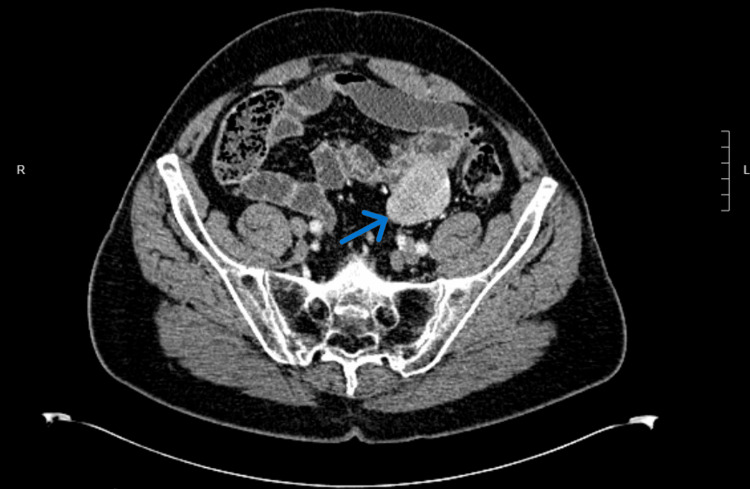
CT enterography three years after hiatal hernia repair showing a small bowel mass.

Four years after the initial diagnosis and treatment of the gastric GIST, the patient was undergoing a diagnostic evaluation for iron deficiency anemia, which was diagnosed after the patient was found to have low hemoglobin and iron levels during his yearly routine physical examination. He was managed with oral iron supplements and underwent regular physical and laboratory monitoring to ensure response to treatment. Upper endoscopy and colonoscopy were unremarkable and a subsequent CT enterography was performed. That study revealed a 5.5 cm solid, mildly hypervascular mass in the left lower quadrant that appeared to be contiguous with the small bowel (Figure 2).

The patient denied abdominal symptoms, such as changes in his bowel habits, abdominal pain, or unexplained weight loss. A laparoscopic resection of the jejunal mass with a small bowel resection was performed, and the recovery was uneventful. Surgical pathology results revealed a spindle-type 3.2 cm GIST with slight mucosal ulceration. Section through mass revealed pale-tan homogenous and striated glistering cut surface with fish fresh appearance. The neoplastic cells were immunoreactive for C-KIT and CD34 but negative for S100 and smooth muscle actin (SMA). The tumor had a histologically low-grade mitotic rate of one per 5 mm2. A restaging CT scan of the abdomen and pelvis revealed no evidence of lymph node involvement or distant metastasis (AJCC pT2N0M0, Stage I). This spindle-type GIST appeared histologically different from his original gastric GIST, which was of the epithelioid subtype. This case was discussed at our institution’s Multidisciplinary Cancer Conference, and it was the consensus opinion that the role of systemic therapy was not well defined in this situation, and since the pathology evaluation suggested low-risk biology, observation with serial imaging was recommended. The patient is currently being seen as an outpatient biannually with CT imaging for surveillance, and after a year remains without evidence of recurrence.

## Discussion

Data from the Surveillance, Epidemiology, and End Results (SEER) database suggest that the annual age-adjusted incidence of GISTs has risen from 0.55 per 100,000 population in 2001 to 0.78 per 100,000 in 2011 [8]. They seem to be more common among non-Hispanics than Hispanics and more in African Americans than whites [8]. GISTs seem to have a slightly higher preponderance in males than females and the median age at diagnosis is between 55 and 65 years [1]. Over the past few decades, there have been improvements in the understanding of the biology of GISTs, with recognition of interstitial cells of Cajal as the likely precursor cells and identification of the mutation in C-KIT (CD117) and platelet-derived growth factor receptor α (PDGRF-α) [9]. Pathologically, diagnosis of a GIST relies on histologic morphology as well as immunochemistry, which highlights C-KIT (CD117) and/or discovered on GIST1 (DOG1) [10].

In a large study evaluating anatomical locations of 9747 GISTs, gastric location was the most frequent (55.6%), followed by small bowel (31.8%), colorectal (6.0%), and esophagus (0.7%) [11]. While GISTs represent a rare class of tumors, their clinical management is complicated by significant heterogeneity, not only anatomically but also at a genetic level. Approximately 85% of GISTs are characterized by mutations in the KIT or PDGFRA genes, which influence cellular signaling pathways critical for tumor growth and survival [12]. Metachronous GISTs, although rare, are particularly challenging as they may develop from independent genetic mutations, leading to varied histological profiles [13]. Primary extra-gastrointestinal stromal tumors have been described in the mesentery, omentum, gallbladder, bladder wall, ovary, rectovaginal septum, and uterus [14]. Histologically, GISTs are classified as spindle cell type (70%), epithelioid cell type (20%), or mixed (10%) [3].

Approximately 18% of all GIST cases are asymptomatic and are detected incidentally during endoscopy, abdominal computer tomography (CT) scans, or unrelated surgical procedures [11,15,16]. Symptoms tend to be non-specific, such as nausea, vomiting, abdominal distension, early satiety, abdominal pain, anemia symptoms (fatigue, angina, dyspnea on exertion, etc.), and rarely as a palpable mass [13]. Our patient’s two presentations highlight the differing ways that GISTs may be diagnosed. His first presentation of a gastric GIST was a purely incidental finding during laparoscopic hiatal hernia repair and was not seen on preoperative endoscopy and CT imaging. His second presentation of a jejunal GIST was detected on CT enterography and was associated with anemia. This patient underwent R0 laparoscopic resections of both an epithelioid-subtype gastric GIST and a spindle-cell subtype jejunal GIST, separated by four years. To the best of our knowledge, this is the first report of a patient presenting with two metachronous primary GISTs demonstrating different pathologic subtypes.

## Conclusions

While metachronous GISTs are relatively rare, the occurrence of such tumors with distinct histological subtypes in the same patient, as demonstrated in this case, underscores a significant gap in our understanding of GIST biology and progression. Despite advances in genetic profiling and therapeutic management of GISTs, the mechanisms driving the development of metachronous tumors with different histologies remain poorly understood. This case highlights the critical need for further longitudinal and comparative studies that can elucidate the underlying genetic and molecular pathways involved in such presentations. Given the potential for distinct biological behavior and therapeutic response to GISTs based on histological subtypes, we propose the development of tailored surveillance strategies that take into account the specific histological characteristics of the tumors. For instance, more frequent imaging intervals may be warranted for patients known to have aggressive histological features or when a previous GIST was of an aggressive subtype. Additionally, there is a need to explore the role of novel molecular markers in predicting the occurrence of metachronous tumors, which could lead to more personalized monitoring and treatment plans.
